# An Expert Diagnosis System for Parkinson Disease Based on Genetic Algorithm-Wavelet Kernel-Extreme Learning Machine

**DOI:** 10.1155/2016/5264743

**Published:** 2016-05-05

**Authors:** Derya Avci, Akif Dogantekin

**Affiliations:** ^1^Department of Electrical and Electronic Engineering, Engineering Faculty, Firat University, 23119 Elazig, Turkey; ^2^Emek Hospital, Gaziantep, Turkey

## Abstract

Parkinson disease is a major public health problem all around the world. This paper proposes an expert disease diagnosis system for Parkinson disease based on genetic algorithm- (GA-) wavelet kernel- (WK-) Extreme Learning Machines (ELM). The classifier used in this paper is single layer neural network (SLNN) and it is trained by the ELM learning method. The Parkinson disease datasets are obtained from the UCI machine learning database. In wavelet kernel-Extreme Learning Machine (WK-ELM) structure, there are three adjustable parameters of wavelet kernel. These parameters and the numbers of hidden neurons play a major role in the performance of ELM. In this study, the optimum values of these parameters and the numbers of hidden neurons of ELM were obtained by using a genetic algorithm (GA). The performance of the proposed GA-WK-ELM method is evaluated using statical methods such as classification accuracy, sensitivity and specificity analysis, and ROC curves. The calculated highest classification accuracy of the proposed GA-WK-ELM method is found as 96.81%.

## 1. Introduction

Parkinson disease (PD) is a degenerative disorder of the central nervous system. It results from the death of dopamine generating cells in the substantia nigra, a region of the mid-brain. This disease affects about 1% of the world population over the age of 55 [1, 2].

In advanced stages of the disease, nonmotor features, such as dementia and dysautonomia, occur frequently [3]. PD is diagnosed in case of presence of two or more cardinal motor features such as rest tremor, bradykinesia, or rigidity [4]. Functional neuroimaging holds the promise of improved diagnosis and allows assessment in early disease [5].

The main symptoms of PD are bradykinesia, tremor, rigidity, and postural instability. When all of these symptoms are seen, then the person can be diagnosed by doctors with Parkinson disease. The dysphonia is considered to be one of the most difficult aspects of Parkinson disease by many patients and their families. Nearly 9 out of 10 people with PD have a speech or voice disorder. Dysphonic symptoms typically include reduced loudness, breathiness, roughness, decreased energy in the higher parts of the harmonic spectrum, and exaggerated vocal tremor and these symptoms can be detected using many different vocal tests [6]. In the design of an automatic diagnosis system for PD, it is more suitable to use the voice data because it is one of the most common symptoms. In literature, there are many studies on speech measurement for general voice disorders [6–12]. In these studies, the speech signals are recorded and then these signals are detected by means of different methods certain properties of these signals. Then, a classifier is used to diagnose patients with PD from certain properties of signal. The classifier is the heart of the automatic diagnosis system. The reliable classifier should diagnose the disease at as high accuracy as possible even though there are many uncontrolled variations. In literature, different classifiers have been proposed for automatic diagnosis of PD. The NNs and adaptive neurofuzzy classifier with linguistic hedges (ANFIS-LH) are investigated for automatic diagnosis of PD in [13]. The performance of probabilistic neural network (PNN) for automatic diagnosis of PD is evaluated in [14]. SVM classifier is also investigated for the same goal in [15]. NNs have some drawbacks such as need of long training time and uncertainties in activation function to be used in hidden layer, number of cells in hidden layer, and the number of hidden layer. In case of SVM, type of kernel function and penalty constant and so forth affects the classification performance. If these parameters are not appropriately selected, the classification performance of SVM degrades. Similarly, the performance of ANFIS depends on type and parameters of membership function and output linear parameters.

Among these classifiers, NNs have been widely used in pattern recognition and regression. The NNs are commonly trained by backpropagation based on a gradient-based learning rule [16]. Up to now, the gradient-based learning methods have been widely applied for learning of NNs [17, 18]. However, they have several shortcomings such as difficult setting of learning parameters, slow convergence, training failures due to local minima, and repetitive learning to improve performance of NNs. Also, it is clear that gradient descent-based learning methods are generally very slow [20].

Recently, the Extreme Learning Machine (ELM) proposed by Huang et al. has been widely used in classification regression problems because of its properties fast learning capability and fast learning. Although output weights are analytically calculated, there is no rule in determination of number of hidden neurons, activation function. The ELM may not provide high classification performance because of cases mentioned above.

In [6], the GA is used for selection of feature subset for the input of ANN. The proposed method is not suitable for real-time implementation. Besides, the feature vector is randomly reduced to a lower dimension in [3, 6, 7, 9]. ANFIS structure might not have a good performance if a huge amount of data exists.

Recently, a new learning algorithm called Extreme Learning Machine (ELM) which randomly selected all the hidden nodes parameters of generalized single-hidden layer feedforward networks (SLFNs) and analytically determines the output weights of SLFNs is proposed in [18–20]. Although output weights are analytically calculated, there is no rule in determination of number of hidden neurons and type of the kernel function. To obtain a good classification performance of ELM, these parameters should be determined properly.

This paper proposes an expert Parkinson diseases (PD) diagnosis system based on genetic algorithm- (GA-) wavelet kernel- (WK-) Extreme Learning Machines (ELM). The classifier used in this paper is single layer neural network (SLNN) and it is trained by the ELM learning method. In wavelet kernel-Extreme Learning Machine (WK-ELM) structure, there are three adjustable parameters of wavelet kernel. These parameters and the numbers of hidden neurons play a major role in the performance of ELM. Therefore, values of these parameters and numbers of hidden neurons should be tuned carefully based on the solved problem. In this study, the optimum values of these parameters and the numbers of hidden neurons of ELM were obtained by using a genetic algorithm (GA). The hepatitis disease datasets are obtained from the UCI machine learning database. The performance of the proposed GA-WK-ELM method is evaluated through statical methods such as classification accuracy, sensitivity and specificity analysis, and ROC curves. In here, the numbers of hidden neurons of ELM and parameters of wavelet kernel function are optimized by GA. In GA structure, an individual is composed of a total of 20 bits. These are as follows:

(i) The first four bits (1st, 2nd, 3rd, and 4th bits) of each of these individuals represent the *w* parameter values (between 1 and 16) of the wavelet kernel functions.
(ii) The second four bits (5th, 6th, 7th, and 8th bits) of each of these individuals represent the *x* parameter values (between 1 and 16) of the wavelet kernel functions.
(iii) The third four bits (9th, 10th, 11th, and 12th bits) of each of these individuals represent the *y* parameter values (between 1 and 16) of the wavelet kernel functions.
(iv) The rest of the 20 bits represent the number of hidden neurons (between 5 and 260).

The 40 number of these individuals is randomly chosen for the initial population. Thus, it is purposed to obtain the best possible performance from ELM classifier. The training and testing dataset for the proposed method is obtained from the UCI dataset. This dataset is composed of 192 pieces of data. The randomly selected 128 of 192 pieces of data are used for training of classifier whereas the remaining data is used for testing of classifier. For different kernel function and number of hidden neurons, the results of the proposed method are given. Further, a comparison is performed with previous studies to show the validity of the proposed method. From results, the proposed method is a quite powerful tool for automatic diagnosis of hepatitis and may work in real-time systems.

The paper is organized as follows.  Section 2 presents pattern recognition for the diagnosis of Parkinson disease. In Section 3, wavelet kernel-Extreme Learning Machines and in Section 4 genetic algorithms are briefly presented, respectively. In Section 5, application of GA-WK-ELM for the diagnosis of Parkinson disease is explained. The obtained results are given in Section 6. Finally, Section 7 provides the discussion and conclusion of this study.

## 2. Pattern Recognition for Diagnosis of Parkinson Disease

The pattern recognition for diagnosis of disease is commonly composed of two stages. They are feature extraction and classification stages. In the feature extraction stage, the useful information of data is extracted by a feature extractor. The feature extraction not only reduces the computational burden of the classifier but also improves classification performance. In classification stage, extracted features from data are given as input to the classifier. Depending on classification problem, the data is separated into two or more classes by the classifier.

**Figure 1:**
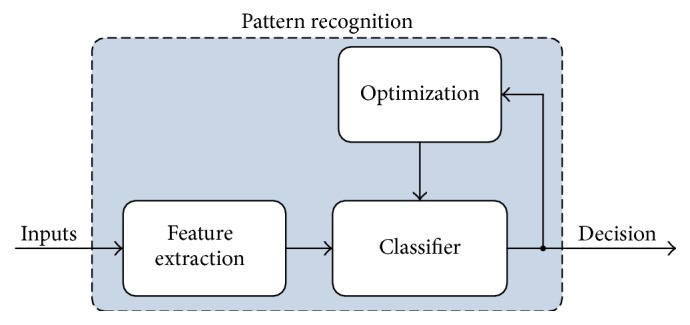
The block diagram of the pattern diagnosis concept.

The pattern recognition concept used in this study is given in Figure 1. The proposed concept consists of three stages including feature extraction, classification, and optimization of classifier's parameters. These stages are explained in detail as follows.

## 3. Wavelet Kernel-Extreme Learning Machines

In literature, the neural networks have been commonly used in pattern recognition and regression problems [20, 21]. The gradient-based learning and backpropagation algorithms are most commonly used methods for neural networks [17, 18]. Moreover, these methods have some drawbacks such as difficult setting of learning parameters, slow convergence, slow learning, and training failures [19].

**Figure 2:**
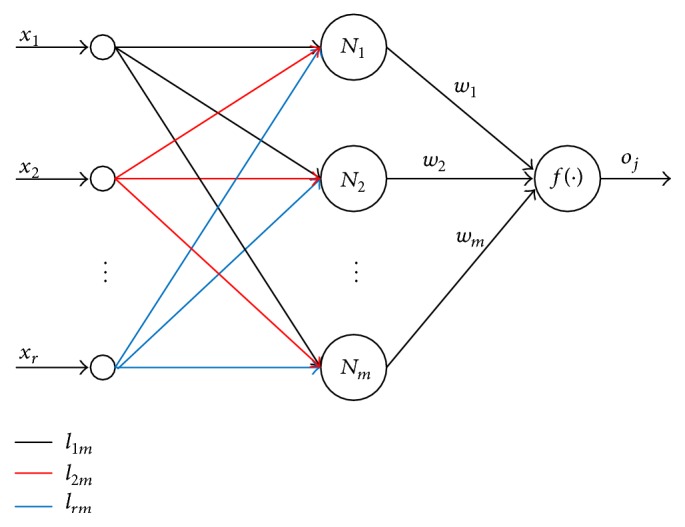
The structure of a single-hidden layer feedforward network.

Because of these disadvantages of classic gradient-based learning and backpropagation neural network algorithms, the Extreme Learning Machine (ELM) algorithm is proposed by Cho et al. [19]. In the Extreme Machine Learning algorithm, the output weights of a single-hidden layer feedforward network (SLFN) are analytically calculated by using the Moore-Penrose (MP) generalized inverse instead of iterative learning scheme [20]. In Figure 2, the structure of a single-hidden layer feedforward network using Extreme Learning Machine algorithm is given. In here, *l*_1*m*_ ,  *l*_2*m*_,  and *l*_*rm*_ are weights vector connecting the *k*th hidden neuron and the input neurons, *w* is the weight vector connecting the *k*th hidden neuron and output neuron, and *f*(·) is the activation function.

The most significant features of ELM are ordered as below:

- In ELM structure, the learning speed is very fast. Because of this, single-hidden layer feedforward network can be trained by using ELM. Thus, an ELM learning method, which is faster than other classical learning methods, is obtained.
- The obtaining of the less training error and the fewer norms of weights are aimed at by using ELM, because the ELM learning algorithm has good performance for neural networks.
- In the structure of single-hidden layer feedforward network, the ELM learning algorithm is used with nondifferentiable activation functions.
- The easy solutions are tried to get in the ELM structure [19].

The outputs of an ELM with *m* neurons and *f* activation function are given as below:

(1) $$\begin{array}{c} o_{j}=\overset{m}{\underset{i=1}{\sum }}\beta_{i}f\left(\;l_{i}x_{r}+b_{i}\;\right). \end{array}$$

The ELM learning algorithm has faster learning speed than classic neural networks. Moreover, it has better generalization performance than them. Nowadays, the number of researchers, who work in ELM science topic, has increased [19–23]. In ELM learning algorithm, the initial parameters of the hidden layer need not be tuned. In ELM algorithm, all nonlinear piecewise continuous functions are used as the hidden neurons. Therefore, for *M* optional various samples {(*r*_*j*_, *m*_*j*_)∣*r*_*j*_ ∈ *Q* ^*l*^, *m*_*j*_ ∈ *Q* ^*k*^, *j* = 1,…, *M*}, the output function in ELM by using *K* hidden neurons is

(2) $$\begin{array}{c} u_{K}\left(\;r\;\right)=\overset{K}{\underset{j=1}{\sum }}S_{j}v_{j}\left(\;r\;\right)=v\left(\;r\;\right)S, \end{array}$$

where *v* = [*v*_1_(*r*), *v*_2_(*r*),…, *v*_*K*_(*r*)]  is the output vector of the hidden layer with respect to the input *r*. *S* = [*S*_1_, *S*_2_,…, *S*_*K*_]  is the vector of the output weights between the hidden layer of *K* neurons and the output neuron. *v* vector changes the data from input space to the ELM feature space [19–23]. The training error and the output weights should be synchronously minimized for decreasing the training error in ELM algorithm. So, generalization performance of neural networks increases:

(3) minimize AS−C, S.

In here, (3) can be solved by using

(4) $$\begin{array}{c} S=A^{T}{\left(\;\frac{1}{E}+AA^{T}\;\right)}^{-1}C, \end{array}$$

where *E* is the regulation coefficient, *A* is the hidden layer output matrix, and *C* is the expected output matrix of samples, respectively. So, the output function of the ELM learning algorithm can be given as follows:

(5) $$\begin{array}{c} u\left(\;r\;\right)=v\left(\;r\;\right)A^{T}{\left(\;\frac{1}{E}+AA^{T}\;\right)}^{-1}C. \end{array}$$

If the feature vector *v*(*r*) is unknown, the kernel matrix of ELM based on Mercer's conditions can be computed as follows:

(6) $$\begin{array}{c} D=AA^{T}:k_{jz}=v\left(\;r_{j}\;\right)v\left(\;r_{z}\;\right)=b\left(\;r_{j},r_{z}\;\right). \end{array}$$

In this way, the output function *u*(*r*) of the wavelet kernel-Extreme Learning Machine (WK-ELM) can be given as below:

(7) $$\begin{array}{c} u\left(\;r\;\right)=\left[\;b\left(\;r,r_{1}\;\right),\ldots ,b\left(\;r,r_{M}\;\right)\;\right]{\left(\;\frac{1}{E}+D\;\right)}^{-1}C. \end{array}$$

In there, *D* = *AA* ^*T*^ and *b*(*r*, *g*) is the kernel function of Extreme Learning Machine. There are some kernel functions, which are linear kernel, polynomial kernel, Gaussian kernel, and exponential kernel, appropriate for the Mercer condition in ELM literature. The readers can find more details in [21, 22]. In this study, wavelet kernel function is used for simulation and performance analysis of WK-ELM:

(8) $$\begin{array}{c} b\left(\;r,g\;\right)=\cos\left(\;w\frac{\left\|r-g\right\|}{x}\;\right)\exp\left(\;-{\frac{\left\|r-g\right\|}{y}}^{2}\;\right). \end{array}$$

In the result of these application studies, it was observed that the training and testing performance of the wavelet kernel function shown in (8) is better than the performances of linear kernel, polynomial kernel, Gaussian kernel, and exponential classical kernel functions, respectively. The values of adjustable parameters *w*,  *x*, and *y* are important for training performance of ELM. Because of this, values of these parameters should be attentively tuned for solving the problem. However, the hidden layer feature mapping need not be known and the number of hidden neurons need not be chosen in WK-ELM algorithms. Moreover, the WK-ELM learning algorithm has better generalization performance than classic ELM learning algorithm. At the same time, it was shown that WK-ELM is more stable than classic ELM and is faster than Support Vector Machine (SVM) [24].

## 4. Genetic Algorithms

To solve a problem, an evolutionary process is used in the structures of genetic algorithms [25]. A genetic algorithm begins with a set of solutions which are represented by individuals. This set of solutions is known as a population. Each population is a solution set and new solutions are selected according to their fitness values. In the genetic algorithm, the iterative process is repeated as long as the new population is better than the old one. The higher the fitness value of an individual is, the more likely this individual is reproduced for the next population. The iterative process is finished when some conditions (e.g., number of individuals in the population) are satisfied [26].

The stages of the genetic algorithm are given as below.

**Stage 1 .**

A random population of *n* individuals is created. These individuals are a suitable solution to the problem. In here, the value of *n* is 20.

**Stage 2 .**

The fitness *f*(*x*) of each individual *x* is calculated in the population [25]. In these experimental studies, each of the individuals in the population is randomly formed.

**Stage 3 .**

Two parental individuals from among the individuals are selected. These individuals have the higher fitness value in the population. Then, the crossover operator is realized to these parental individuals. The aim of the crossover operator is creating the varied individuals. These have higher fitness values than former individuals.

**Stage 4 .**

In this stage, a crossover probability is used for crossover operating to form the new individuals. If crossover is not done, the individual will be the exact copy of the parents.

**Stage 5 .**

In this stage, each new individual is obtained by mutating with a mutation probability. This mutation process is realized by using any one or more bits of the individual.

**Stage 6 .**

In this stage, the new individuals are obtained from the new population.

**Stage 7 .**

In this stage, if the end conditions are satisfied, the genetic algorithm is stopped. It is returned to the best solution in the current population.

**Stage 8 .**

In this stage, it is returned to Stage 2. Then, the new generated population is used for further algorithm.

## 5. Application of GA-WK-ELM for Diagnosis of Parkinson Disease

The Parkinson dataset used in this study is composed of a range of biomedical voice measurements from 31 people, 23 with Parkinson disease (PD), and it includes a total of 192 voice recordings from individuals. In addition, these biomedical voice measurements have different attribute information given in Table 1 [6–12].

The essential aim of processing the data is to discriminate healthy people from those with PD, according to the “status” attribute which is set to 1 for healthy people and 0 for people with PD, which is a two-decision classification problem.

**Figure 3:**
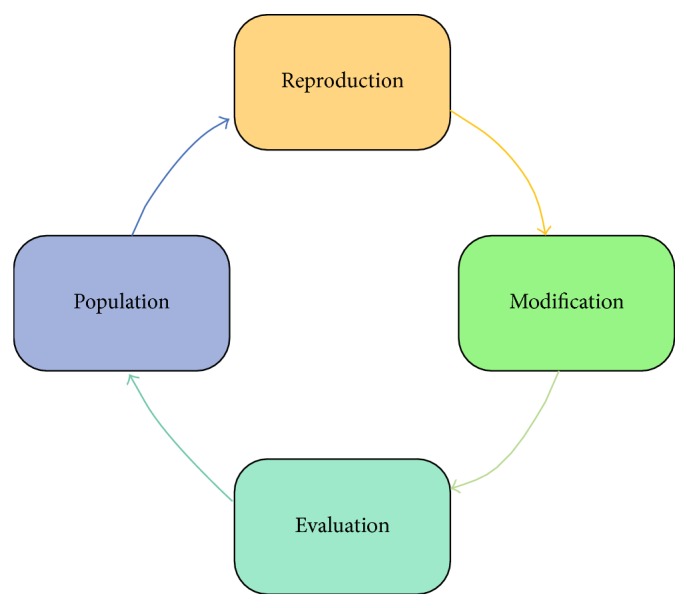
Components of a GA.

The block diagram of the proposed method is given in Figure 3. As shown in the figure, the feature vector obtained from the PD dataset is applied to WK-ELM optimized with GA. The Parkinson dataset used in this study is taken from the University of California at Irvine (UCI) machine learning repository [6–12]. It was used for training and testing of the proposed GA-WK-ELM method.

The dataset has 22 relevant features as given in Table 1 and includes a total of 192 cases. Thus, it is a matrix with dimensions of 192 × 22. Training of the GA-ELM is carried out with dataset of 128 and the remaining data is used for testing of GA-WK-ELM. To optimize the parameters of WK-ELM, GA is used. The fitness function of the GA is training error of WK-ELM classifier.

This GA-WK-ELM method for diagnosis of PD includes three layers. In the first layer of GA-WK-ELM, the Parkinson data is obtained from the UCI machine learning database mentioned in Section 5. In the second layer of GA-WK-ELM, the numbers of hidden neurons of WK-ELM and parameters of wavelet kernel function are optimized by the GA. In the GA structure, an individual has a total of 20 bits. These bits can be ordered as below:

(i) *The First Four Bits of GA-WK-ELM*. In this structure of GA-WK-ELM, the 1st, 2nd, 3rd, and 4th bits of each of these individuals symbolize the *w* parameter values (between 1 and 16) of the wavelet kernel functions.
(ii) *The Second Four Bits of GA-WK-ELM*. In this structure of GA-WK-ELM, the 5th, 6th, 7th, and 8th bits of each of these individuals symbolize the *x* parameter values (between 1 and 16) of the wavelet kernel functions.
(iii) *The Third Four Bits of GA-WK-ELM*. In this structure of GA-WK-ELM, the 9th, 10th, 11th, and 12th bits of each of these individuals symbolize the *y* parameter values (between 1 and 16) of the wavelet kernel functions.
(iv) *The Rest of the Bits of GA-WK-ELM*. In this structure of GA-WK-ELM, the rest of the 20 bits symbolize the number of hidden neurons (between 5 and 260).

The 40 number of these individuals is randomly selected for the initial population. So, this GA structure is purposed to obtain the best possible performance from the WK-ELM classifier. The training and testing dataset for the proposed GA-WK-ELM method is obtained from the UCI dataset. This Parkinson disease dataset includes 192 pieces of data. The randomly selected 100 of 192 pieces of PD data are used for training of WK-ELM classifier. The rest of this PD data is used for testing of the WK-ELM classifier. In here, the results of the proposed GA-WK-ELM method are given for the optimum parameters values of wavelet kernel function and number of hidden neurons of WK-ELM. In here, a comparison is performed with previous studies to show the validity of the proposed GA-WK-ELM method. From results, the proposed GA-WK-ELM method is a quite powerful tool for automatic diagnosis of hepatitis and may work in real-time systems.

**Figure 4:**
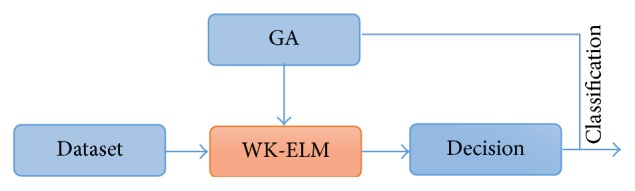
The block diagram of GA-WK-ELM based optimal PD diagnosis system.

The block diagram of the proposed GA-WK-ELM method is given in Figure 4. In these applications, a 3-fold cross-validation schema was applied where the two-fifths data were used for training the proposed GA-WK-ELM method and the other remaining data were used as the test dataset. This method was repeated for three times for obtaining the average classification rates. Thus, the correct diagnosis performance of the suggested GA-WK-ELM method is computed.

In here, the maximum training accuracy value of the WK-ELM classifier was used as the fitness function of GA. This maximum training accuracy was calculated from the result of training of WK-ELM for each of the individuals by using parameters represented by these individuals. The *w*,  *x*,  and *y* parameters values of wavelet kernel function and the number of hidden neurons of the WK-ELM classifier are optimized by GA. The PD dataset has 22 relevant features. These features were obtained from 192 patients. So, dimensions of the features vector are 192 × 22. Here, 40 random individuals are selected as the initial population. Each of these individuals has 20 bits. In Tables 2 and 3, coding for parameters of wavelet kernel function and the number of hidden neurons are given, respectively.

**Figure 5:**
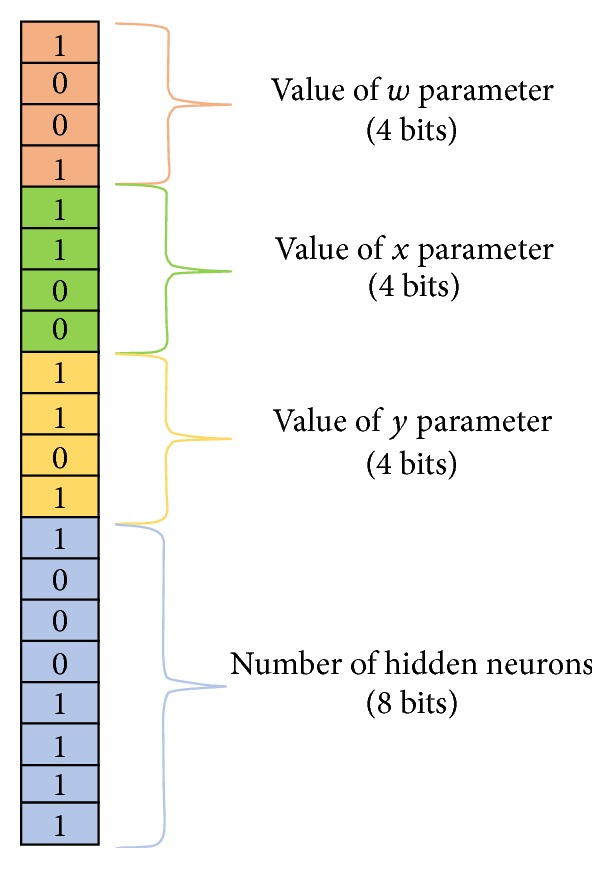
An example for individuals of the population.

An example for individuals of the population is shown in Figure 5. The 1st, 2nd, 3rd, and 4th bits of this individual symbolize the *w* parameter values of the wavelet kernel functions, which are between 1 and 16. The 5th, 6th, 7th, and 8th bits of this individual symbolize the *x* parameter values of the wavelet kernel functions, which are between 1 and 16. The 9th, 10th, 11th, and 12th bits of this individual symbolize the *y* parameter values of the wavelet kernel functions, which are between 1 and 16. The rest of the 20 bits of this individual symbolize the number of hidden neurons, which are between 5 and 260.

The correct diagnosis performance of the suggested GA-WK-ELM method for PD dataset is computed by three evaluation methods as classification accuracy, sensitivity and specificity analysis, and Receiver Operating Characteristic (ROC) curves.

The performance of the proposed method is evaluated by the* Sensitivity Analysis* (SEA) and* Specificity Analysis* (SPA) and classification accuracies, which are obtained from statistical methods in given equations ((9)–(11)), respectively, are presented in Table 6:

(9) SEA=the number of correctly classified persons with PDthe number of total PD cases,

(10) SPA=the number of persons correctly classified as healthythe number of total healthy persons.

The overall classification correct ratio of the proposed method (OC) is calculated as (7):

(11) $$\begin{array}{c} \text{OC}=\frac{\text{the number of correct classifications}}{\text{the number of total cases}}. \end{array}$$

In this experimental study, a genetic algorithm structure was designed for deciding the *w*, *x*, *y* parameters values of wavelet kernel function and the number of hidden neurons of the WK-ELM classifier. A total of 20 bits are used for each of the individuals in the initial population. In this GA structure, the 1st, 2nd, 3rd, and 4th bits of the individual give the *w* parameter value, the 5th, 6th, 7th, and 8th bits of this individual give the *x* parameter value, and the 9th, 10th, 11th, and 12th bits of the individual give the *y* parameter value, respectively. The remainder of the 20 bits of the individual give the number of hidden neurons, which are between 5 and 260.

## 6. Obtained Results

In these experimental studies, an expert diagnosis system for PD based on the GA-WK-ELM method is introduced. Then, the correct PD diagnosis performance of the suggested GA-WK-ELM method is also evaluated by classification accuracy, sensitivity and specificity analysis, and ROC curve, respectively.

The suggested GA-WK-ELM method is used for finding the optimum values of the wavelet kernel function *w*,  *x*,  and *y* parameters and the number of ELM classifier hidden neurons in these experimental studies. The comparing results by using the GA-WK-ELM method and the classic ELM classifier by using the same PD database can be given in Table 4. In these classic ELM classifiers, each of sigmoid, tangent sigmoid, triangular basis, radial basis, hard limit, and polykernel functions is used as the kernel function, respectively. The readers can find more detailed information about these kernel functions in [21, 22]. As shown in this table, the best correct diagnosis rate of the suggested GA-WK-ELM method is found as 96.81% by using 15, 3, 10, and 86 values for the *w*, *x*, *y* wavelet kernel function parameters and the number of hidden neurons, respectively.

As shown in Table 4, the highest correct PD diagnosis rate is obtained as 96.81% by using the suggested GA-WK-ELM method, because the optimum values of the WK-ELM *w*,  *x*, *y* parameters and the numbers of hidden neurons of WK-ELM were obtained by using a genetic algorithm (GA) in these experimental studies. In this study, after finding the optimum parameters, we do not need to use GA and then the WK-ELM can be directly used.

In Table 5, to show the validity of the suggested GA-WK-ELM method, compared results with previous studies using the same dataset [6–12] are also given. From this table, the highest diagnosis rate is calculated as 94.72 by [13] by using the neurofuzzy classifier with linguistic hedges (ANFIS-LH). In here, training times have not been given in these studies. Moreover, the feature vector is randomly reduced to a lower dimension in [13]. The suggested GA-WK-ELM method in this study shows a correct diagnosis performance even though the feature vector is directly used without reduction. However, the training time of WK-ELM is extremely short.

The obtained PD diagnosis accuracies by statistical evaluation criteria are given in Table 6.

In this study, ROC curves and AUC values are calculated by using TP, TN, FP, and FN, which are true positives, true negatives, false positives, and false negatives, respectively [27]. The used ROC curve in this study is a graphical plot. It shows the performance of a binary classifier system as its discrimination threshold is varied. The ROC curve is formed by plotting the true-positive rate against the false-positive rate at various threshold settings. In here, the true-positive rate is also known as sensitivity in biomedical informatics or recall in machine learning. The false-positive rate is also known as the fallout. It can be calculated as 1 − specificity. So, the ROC curve is the sensitivity as a function of the fallout.

**Figure 6:**
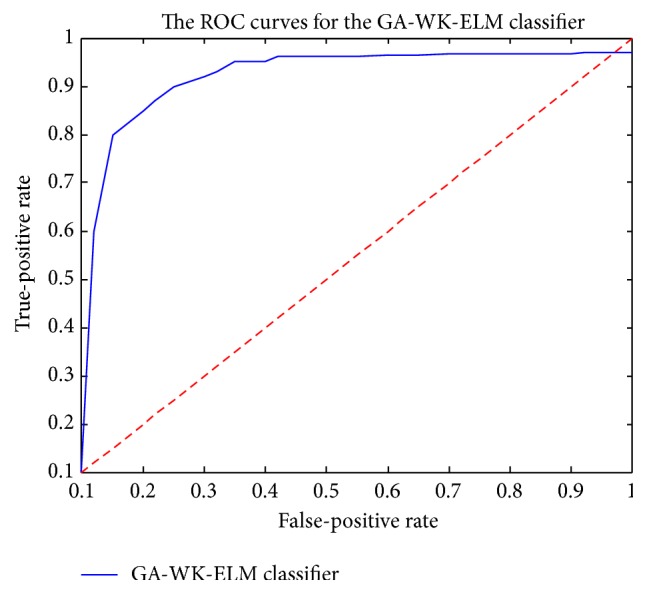
The ROC curve of the suggested GA-WK-ELM method for expert PD diagnosis.

ROC analysis supplies tools to choose the optimal models. Moreover, it eliminates the suboptimal ones independently from the class distribution or the cost context. ROC analysis is related in a direct and natural way to cost/benefit analysis of diagnostic decision-making. In here, the ROC curve of GA-WK-ELM is given by using the obtained best TP, TN, FP, and FN values in Figure 6. The obtained AUC value of ROC curves by using the GA-WK-ELM classifier can be given as 0,9576.

## 7. Discussion and Conclusion

This paper suggests an expert PD diagnosis system based on GA-WK-ELM. The proposed GA-WK-ELM PD diagnosis system has advantages such as finding of the optimal *w*,  *x*,  and *y* parameters combination of wavelet kernel, direct using of feature vector, fast training and testing time, and generalization capability over conventional neural networks with backpropagation. The suggested GA-WK-ELM method is formed from two stages as WK-ELM classification and optimization of WK-ELM classifier's parameters. The feature vector from Parkinson dataset is used as input to the WK-ELM classifiers. In wavelet kernel-Extreme Learning Machine (WK-ELM) structure, there are three adjustable *w*,  *x*,  and *y* parameters of wavelet kernel. These *w*,  *x*,  and *y* parameters and the numbers of hidden neurons play a major role in the performance of WK-ELM. Because of this, values of these *w*,  *x*,  and *y* parameters and numbers of hidden neurons should be carefully set based on the solved diagnosis of the PD problem. In this paper, the optimum values of these wavelet kernel parameters and the numbers of hidden neurons of WK-ELM were calculated by using GA. The output of WK-ELM makes decisions about diagnosis of PD. The optimum values of the wavelet kernel *w*,  *x*,  and *y* parameters and numbers of hidden neurons of the WK-ELM classifier are calculated by a GA to obtain the best possible PD diagnosis performance. The feasibility of the suggested GA-WK-ELM method has been tested by using PD dataset. This dataset has 192 test cases. The suggested GA-WK-ELM method has effective PD diagnosis performance when compared with previous studies depending on direct using of the same feature vector and training time as shown in Tables 4 [Table tab5]–6 and Figure 6.

## Figures and Tables

**Table 1:** The attributes of biomedical voice measurements.

Number	Attributes	Explanation
1	MDVP:Fo (Hz)	Average vocal fundamental frequency

2	MDVP:Fhi (Hz)	Maximum vocal fundamental frequency

3	MDVP:Flo (Hz)	Minimum vocal fundamental frequency

4	MDVP:Jitter (%)	Several measures of variation in fundamental frequency
5	MDVP:Jitter (abs)	
6	MDVP:RAP	
7	MDVP:PPQ	
8	Jitter:DDP	

9	MDVP:Shimmer	Several measures of variation in amplitude
10	MDVP:Shimmer (dB)	
11	Shimmer:APQ3	
12	Shimmer:APQ5	
13	MDVP:APQ	
14	Shimmer:DDA	

15	RPDE	Two nonlinear dynamical complexity measures
16	D2	

17	NHR	The measure of ratio of noise to tonal components in the voice status
18	HNR	

19	DFA	Signal fractal scaling exponent

20	spread1	Three nonlinear measures of fundamental frequency variation
21	spread2	
22	PPE	

**Table 2:** Coding for parameters of wavelet kernel function.

Values of *w*, *x*, and *y* parameters	Coding
1	$\begin{array}{cccc} 0 & 0 & 0 & 0 \end{array}$
2	$\begin{array}{cccc} 0 & 0 & 0 & 1 \end{array}$
3	$\begin{array}{cccc} 0 & 0 & 1 & 0 \end{array}$
4	$\begin{array}{cccc} 0 & 0 & 1 & 1 \end{array}$
5	$\begin{array}{cccc} 0 & 1 & 0 & 0 \end{array}$
6	$\begin{array}{cccc} 0 & 1 & 0 & 1 \end{array}$
7	$\begin{array}{cccc} 0 & 1 & 1 & 0 \end{array}$
8	$\begin{array}{cccc} 0 & 1 & 1 & 1 \end{array}$
9	$\begin{array}{cccc} 1 & 0 & 0 & 0 \end{array}$
10	$\begin{array}{cccc} 1 & 0 & 0 & 1 \end{array}$
11	$\begin{array}{cccc} 1 & 0 & 1 & 0 \end{array}$
12	$\begin{array}{cccc} 1 & 0 & 1 & 1 \end{array}$
13	$\begin{array}{cccc} 1 & 1 & 0 & 0 \end{array}$
14	$\begin{array}{cccc} 1 & 1 & 0 & 1 \end{array}$
15	$\begin{array}{cccc} 1 & 1 & 1 & 0 \end{array}$
16	$\begin{array}{cccc} 1 & 1 & 1 & 1 \end{array}$

**Table 3:** Coding for number of hidden neurons.

The number of hidden neurons	Coding
5	$\begin{array}{cccccccc} 0 & 0 & 0 & 0 & 0 & 0 & 0 & 0 \end{array}$
6	$\begin{array}{cccccccc} 0 & 0 & 0 & 0 & 0 & 0 & 0 & 1 \end{array}$
7	$\begin{array}{cccccccc} 0 & 0 & 0 & 0 & 0 & 0 & 1 & 0 \end{array}$
8	$\begin{array}{cccccccc} 0 & 0 & 0 & 0 & 0 & 0 & 1 & 1 \end{array}$
⋮	⋮
260	$\begin{array}{cccccccc} 1 & 1 & 1 & 1 & 1 & 1 & 1 & 1 \end{array}$

**Table 4:** The correct Parkinson diseases diagnosis performance comparing of the GA-WK-ELM method with classic ELM classifiers, which have different types of kernel function and the number of hidden neurons.

Used method	Type of the kernel function	Value of *w* wavelet kernel parameter	Value of *x* wavelet kernel parameter	Value of *y* wavelet kernel parameter	The number of hidden neurons	Accuracy (%)
GA-WK-ELM	Wavelet	6	5	12	74	96.32
*GA-WK-ELM*	*Wavelet*	*15*	*3*	*10*	*86*	*96.81*
GA-WK-ELM	Wavelet	9	4	17	23	95.46
GA-WK-ELM	Wavelet	5	4	12	42	95.28
Classic ELM	Poly	—	—	—	76	89.31
Classic ELM	Hard limit	—	—	—	142	83.22
Classic ELM	Tangent sigmoid	—	—	—	164	91.75
Classic ELM	Tangent sigmoid	—	—	—	242	91.64
Classic ELM	Poly	—	—	—	56	83.85
Classic ELM	Radial basis	—	—	—	108	87.62
Classic ELM	Sigmoid	—	—	—	265	92.28
Classic ELM	Radial basis	—	—	—	356	91.54
Classic ELM	Radial basis	—	—	—	462	93.45

**Table 5 tab5:** The comparison results of the proposed GA-WK-ELM method and previous studies.

Studies	Method	The number of features	Training		Testing
			Time	Accuracy (%)	Accuracy (%)
Ref [13]	ANFIS-LH	4	—	95.38	94.72
	MLPNN	4	—	93.88	89.69
	RBFNN	4	—	91.84	87.63

Ref [14]	PNN	22	—	81.74	81.28

In this study	GA-WK-ELM	22	0.21 *μ*s	99.42	96.81

**Table 6:** The obtained PD diagnosis accuracy by statistical methods.

Method	Correct diagnosis rate
Sensitivity Analysis	95.45
Specificity Analysis	98.17
Average	96.81

## References

[1] Betarbet R., Sherer T. B., Greenamyre J. T. (2002). Animal models of Parkinson's disease. *BioEssays*.

[2] Wooten G. F., Currie L. J., Bovbjerg V. E., Lee J. K., Patrie J. (2004). Are men at greater risk for Parkinson's disease than women?. *Journal of Neurology, Neurosurgery and Psychiatry*.

[3] Tolosa E., Wenning G., Poewe W. (2006). The diagnosis of Parkinson's disease. *Lancet Neurology*.

[4] Hughes A. J., Daniel S. E., Kilford L., Lees A. J. (1992). Accuracy of clinical diagnosis of diopathic Parkinson's disease: a clinico-pathological study of 100 cases. *British Medical Journal*.

[5] Piccini P. P., Whone A. (2004). Functional brain imaging in the differential diagnosis of Parkinson's disease. *The Lancet Neurology*.

[6] Little M. A., McSharry P. E., Hunter E. J., Spielman J., Ramig L. O. (2009). Suitability of dysphonia measurements for telemonitoring of Parkinson's disease. *IEEE Transactions on Biomedical Engineering*.

[7] Boyanov B., Hadjitodorov S. (1997). Acoustic analysis of pathological voices: a voice analysis system for the screening and laryngeal diseases. *IEEE Engineering in Medicine and Biology Magazine*.

[8] Godino-Llorente J. I., Gómez-Vilda P. (2004). Automatic detection of voice impairments by means of short-term cepstral parameters and neural network based detectors. *IEEE Transactions on Biomedical Engineering*.

[9] Hadjitodorov S., Boyanov B., Teston B. (2000). Laryngeal pathology detection by means of class-specific neural maps. *IEEE Transactions on Information Technology in Biomedicine*.

[10] Rahn D. A., Chou M., Jiang J. J., Zhang Y. (2007). Phonatory impairment in Parkinson's disease: evidence from nonlinear dynamic analysis and perturbation analysis. *Journal of Voice*.

[11] Little M., McSharry P., Moroz I., Roberts S. Nonlinear biophysically-informed speech pathology detection.

[12] Little M. A., McSharry P. E., Roberts S. J., Costello D. A. E., Moroz I. M. (2007). Exploiting Nonlinear recurrence and Fractal scaling properties for voice disorder detection. *BioMedical Engineering Online*.

[13] Caglar M. F., Cetisli B., Toprak I. B. (2010). Automatic recognition of Parkinson's disease from sustained phonation tests using ANN and adaptive neuro-fuzzy classifier. *Journal of Engineering Science and Design*.

[14] Ene M. (2008). Neural network based approach to disriminate healty people from those with Parkinson’s disease. *Annals of University of Craiova: Mathematics and Computer Science Series*.

[15] David G. A., Magnus J. B. (2009). Diagnosing Parkinson by using artificial neural networks and support vector machines. *Global Journal of Computer Science and Technology*.

[16] Sakar C. O., Kursun O. (2010). Telediagnosis of parkinson's disease using measurements of dysphonia. *Journal of Medical Systems*.

[17] Suresh S., Omkar S. N., Mani V. (2005). Parallel implementation of back-propagation algorithm in networks of workstations. *IEEE Transactions on Parallel and Distributed Systems*.

[18] Hsu C. T., Kang M. S., Chen C. S. (2005). Design of adaptive load shedding by artificial neural networks. *IEE Proceedings—Generation, Transmission and Distribution*.

[19] Cho J. H., Lee D. J., Chun M. G. (2007). Parameter optimization of extreme learning machine using bacterial foraging algorithm. *Journal of Fuzzy Logic and Intelligent Systems*.

[20] Huang G.-B., Zhou H., Ding X., Zhang R. (2012). Extreme learning machine for regression and multiclass classification. *IEEE Transactions on Systems, Man, and Cybernetics B*.

[21] Huang W., Li N., Lin Z. Liver tumor detection and segmentation using kernel-based extreme learning machine.

[22] Ding S. F., Zhang Y. A., Xu X. Z., Bao L. N. (2013). A novel extreme learning machine based on hybrid kernel function. *Journal of Computers*.

[23] Huang G.-B., Zhu Q.-Y., Siew C.-K. Extreme learning machine: a new learning scheme of feedforward neural networks.

[24] Li B., Rong X., Li Y. (2014). An improved kernel based extreme learning machine for robot execution failures. *The Scientific World Journal*.

[25] Melanie M. (1999). *An Introduction to Genetic Algorithms*.

[26] Whitley D. (2014). An executable model of a simple genetic algorithm. *Foundations of Genetic Algorithms*.

[27] Huang J., Ling C. X. (2005). Using AUC and accuracy in evaluating learning algorithms. *IEEE Transactions on Knowledge and Data Engineering*.

